# Pharmacogenomics of Antineoplastic Therapy in Children: Genetic Determinants of Toxicity and Efficacy

**DOI:** 10.3390/pharmaceutics18020165

**Published:** 2026-01-27

**Authors:** Zaure Dushimova, Timur Saliev, Aigul Bazarbayeva, Gaukhar Nurzhanova, Ainura Baibadilova, Gulnara Abdilova, Ildar Fakhradiyev

**Affiliations:** 1 Department of Fundamental Medicine, Al-Farabi Kazakh National University, Almaty 050040, Kazakhstan; 2 Institute of Fundamental and Applied Medical Research, S.D. Asfendiyarov Kazakh National Medical University, Almaty 050000, Kazakhstan; fakhradiyev.i@kaznmu.kz; 3 Scientific Centre of Pediatric and Pediatric Surgery, Al-Farabi Avenue 146, Almaty 050060, Kazakhstan; 4 College of Medicine, Korea University, Seoul 02841, Republic of Korea

**Keywords:** pharmacogenomics, pediatric oncology, chemotherapy toxicity, drug efficacy, genetic polymorphisms, *TPMT*, *NUDT15*, *DPYD*, personalized therapy, multi-omics, artificial intelligence

## Abstract

Over the past decades, remarkable progress in multimodal therapy has significantly improved survival outcomes for children with cancer. Yet, considerable variability in treatment response and toxicity persists, often driven by underlying genetic differences that affect the pharmacokinetics and pharmacodynamics of anticancer drugs. Pharmacogenomics, the study of genetic determinants of drug response, offers a powerful approach to personalize pediatric cancer therapy by optimizing efficacy while minimizing adverse effects. This review synthesizes current evidence on key pharmacogenetic variants influencing the response to major classes of antineoplastic agents used in children, including thiopurines, methotrexate, anthracyclines, alkylating agents, vinca alkaloids, and platinum compounds. Established gene–drug associations such as *TPMT*, *NUDT15*, *DPYD*, *SLC28A3*, and *RARG* are discussed alongside emerging biomarkers identified through genome-wide and multi-omics studies. The review also examines the major challenges that impede clinical implementation, including infrastructural limitations, cost constraints, population-specific variability, and ethical considerations. Furthermore, it highlights how integrative multi-omics, systems pharmacology, and artificial intelligence may accelerate the translation of pharmacogenomic data into clinical decision-making. The integration of pharmacogenomic testing into pediatric oncology protocols has the potential to transform cancer care by improving drug safety, enhancing treatment precision, and paving the way toward ethically grounded, personalized therapy for children.

## 1. Introduction

Childhood cancers represent a biologically and clinically distinct group of malignancies compared to those occurring in adults. They differ in cellular origin, genetic landscape, and therapeutic response, often arising from developmental or embryonal tissues rather than from cumulative environmental exposures. Over recent decades, advances in multimodal therapy, including risk-adapted poly-chemotherapy, hematopoietic stem cell transplantation, targeted therapy, and supportive care, have dramatically improved survival rates in pediatric oncology [1,2].

However, despite these achievements, substantial inter-individual variability persists in treatment efficacy and in the incidence and severity of therapy-related toxicities. Even under standardized chemotherapy protocols, some children experience life-threatening adverse effects, while others tolerate intensive therapy with minimal complications [3,4]. These differences are recognized as consequences of genetic variability affecting drug absorption, distribution, metabolism, and elimination.

Pharmacogenomics, the study of how inherited and somatic genetic variations influence drug response, offers a critical framework for understanding this variability. By identifying genetic determinants of both therapeutic efficacy and toxicity, pharmacogenomic research enables the personalization of treatment intensity, the prevention of severe adverse events, and the optimization of clinical outcomes [5]. In pediatric oncology, where dose precision and cumulative toxicity directly influence both survival and long-term quality of life, such insights are of particular importance [6].

This review summarises current knowledge on the pharmacogenomic determinants of antineoplastic drug response in children. It focuses on well-established and emerging biomarkers associated with drug metabolism and toxicity, highlights challenges in implementing genomic testing within clinical workflows, and discusses future directions for integrating pharmacogenomics into precision pediatric oncology.

## 2. Genetic Determinants of Chemotherapy Response and Toxicity

The considerable variability in therapeutic response and treatment-related toxicity among pediatric cancer patients is largely influenced by genetic factors that govern drug metabolism, transport, and cellular sensitivity. Advances in pharmacogenomics have made it possible to identify germline and somatic variants that account for individual differences in both efficacy and adverse event profiles across major classes of antineoplastic agents [7,8]. Understanding these pharmaco-genetic determinants is fundamental for optimizing drug selection, dosing, and overall treatment outcomes in pediatric oncology.

Among the most clinically relevant examples are the antimetabolites, which form the backbone of therapy for acute lymphoblastic leukemia and several other childhood malignancies. Variants in the *TPMT* and *NUDT15* genes significantly affect the metabolism of thiopurines such as 6-mercaptopurine and azathioprine [9] (Table 1). Patients with reduced or absent enzymatic activity accumulate cytotoxic thioguanine nucleotides, predisposing them to severe myelosuppression. Consequently, *TPMT* and *NUDT15* genotyping or phenotyping has been incorporated into many international protocols to guide dose modification and prevent hematologic toxicity [10]. Another widely used antimetabolite, methotrexate, also demonstrates variable pharmacokinetics influenced by genetic polymorphisms. Variants in *MTHFR* (*C677T* and *A1298C*) and *SLCO1B1* affect methotrexate clearance and are associated with an increased risk of mucositis, hepatotoxicity, and neurotoxicity [11]. Although these associations are well documented, the clinical translation of methotrexate pharmacogenomics remains inconsistent across populations, emphasizing the need for validation in ethnically diverse cohorts.

Alkylating agents such as cyclophosphamide and ifosfamide are prodrugs requiring metabolic activation by hepatic cytochrome P450 enzymes [12] (Table 1). Polymorphisms in *CYP2B6* and *ALDH1A1* influence both activation and detoxification processes, thereby altering therapeutic efficacy and the likelihood of dose-limiting toxicities, including cardiotoxicity and hemorrhagic cystitis [13]. In addition, *GSTA1* variants, which affect glutathione S-transferase activity, have been associated with a higher risk of hepatic veno-occlusive disease following high-dose chemotherapy [14].

Anthracyclines, including doxorubicin and daunorubicin, are among the most effective yet cardiotoxic agents used in pediatric oncology [15,16]. Genetic variations in *SLC28A3* and *RARG* have been linked to anthracycline-induced cardiomyopathy. Screening for these variants may enable clinicians to identify high-risk patients and consider cardioprotective strategies such as dexrazoxane co-administration, dose limitation, or schedule modification, balancing efficacy with long-term cardiac safety [17].

Similarly, genetic polymorphisms modulate responses to vinca alkaloids and platinum-based agents, which remain essential components of many pediatric chemotherapy regimens [18]. Reduced *CYP3A5* enzyme activity results in higher vincristine plasma concentrations and an increased risk of peripheral neuropathy, a frequent cause of treatment modification or discontinuation [19]. For cisplatin and related compounds, variants in *ERCC1* and *GSTP1*, key genes involved in DNA repair and detoxification, have been associated with elevated susceptibility to ototoxicity, nephrotoxicity, and neurotoxicity [20]. These findings suggest that pre-treatment genetic screening could help anticipate toxicity and inform dose optimization or protective measures.

Collectively, evidence from pharmacogenomic studies highlights the substantial role of inherited and tumor-specific genetic variation in determining both the efficacy and toxicity of antineoplastic therapy in children. Integrating such information into clinical decision-making could substantially reduce adverse outcomes, improve treatment precision, and enhance survival rates. Nevertheless, the translation of pharmacogenomic discoveries into routine pediatric oncology practice remains limited by small cohort sizes, inconsistent findings, and population heterogeneity. Large, multicenter, and ethnically representative studies are essential to validate genotype–phenotype relationships and establish evidence-based guidelines for the clinical application of pharmacogenomic testing in childhood cancer therapy.

**Table 1:** Genetic determinants of chemotherapy response and toxicity in pediatric oncology.

Drug Class/Agent	Key Genes/Variants	Pharmacogenomic Effect	Clinical Consequences	Potential Clinical Application	Ref.
Antimetabolites (Thiopurines: 6-mercaptopurine, Azathioprine)	*TPMT*, *NUDT15*	Reduced enzymatic activity leads to accumulation of cytotoxic thioguanine nucleotides	Severe myelosuppression, hematologic toxicity	Pre-treatment *TPMT*/*NUDT15* genotyping or phenotyping to guide thiopurine dose adjustment	[21]
Antimetabolites (Methotrexate)	*MTHFR* (C677T, A1298C), *SLCO1B1*	Altered folate metabolism and impaired methotrexate transport	Increased risk of mucositis, hepatotoxicity, neurotoxicity	Genetic testing for *MTHFR* and *SLCO1B1* variants to predict methotrexate clearance and toxicity	[22]
Alkylating Agents (Cyclophosphamide, Ifosfamide)	*CYP2B6*, *ALDH1A1*, *GSTA1*	Variants affect drug activation/detoxification and glutathione conjugation	Cardiotoxicity, hemorrhagic cystitis, hepatic veno-occlusive disease	Genotyping to identify poor metabolizers and adjust dosing or protective regimens	[13]
Anthracyclines (Doxorubicin, Daunorubicin)	*SLC28A3*, *RARG*	Variants influence anthracycline uptake and retinoic acid signaling	Anthracycline-induced cardiomyopathy	Screening for *SLC28A3* and *RARG* variants to identify high-risk patients and guide cardioprotective strategies (e.g., dexrazoxane)	[23]
Vinca Alkaloids (Vincristine)	*CYP3A5*	Reduced enzyme activity increases vincristine plasma levels	Peripheral neuropathy	Genotyping to predict neuropathy risk and adjust dosage	[24]
Platinum Compounds (Cisplatin, Carboplatin)	*ERCC1*, *GSTP1*	Impaired DNA repair and detoxification pathways	Ototoxicity, nephrotoxicity, neurotoxicity	Pre-treatment screening to anticipate toxicity and guide protective interventions	[25]

## 3. Implementation of Pharmacogenomic Testing in Pediatric Oncology

The implementation of pharmacogenomic testing in pediatric oncology represents a critical step toward the realization of precision medicine, yet its integration into routine clinical practice remains inconsistent worldwide. Although numerous gene–drug associations have been identified and validated, translating these discoveries into standardized treatment protocols is challenging due to technical, economic, and systemic barriers [26]. Nonetheless, growing evidence demonstrates that pharmacogenomic-guided therapy can enhance drug safety, optimize efficacy, and improve quality of life for children undergoing cancer treatment.

At present, a limited number of pharmacogenetic markers have achieved sufficient clinical validation to be incorporated into established dosing guidelines. The Clinical Pharmacogenetics Implementation Consortium (CPIC) and the Dutch Pharmacogenetics Working Group (DPWG) have issued recommendations for several gene–drug pairs relevant to pediatric oncology [27]. Among the most widely implemented are *TPMT* and NUDT15 variants for thiopurine dosing in acute lymphoblastic leukemia, *DPYD* variants for fluoropyrimidine toxicity, and *CYP2D6* polymorphisms for drugs such as tamoxifen [28] (Table 2). These guidelines provide clinicians with evidence-based strategies for dose modification or alternative therapy selection based on genotype, thereby reducing the risk of severe toxicity and improving treatment adherence.

Despite such progress, the clinical uptake of pharmacogenomic testing in pediatric settings remains limited. One major obstacle is the lack of large-scale, pediatric-specific studies validating pharmacogenomic associations across diverse populations. Many early studies were conducted predominantly in adult cohorts or small, ethnically homogeneous pediatric samples, limiting the generalizability of findings [29]. Given that allele frequencies for key pharmacogenes, such as *NUDT15*, *SLCO1B1*, or *GSTA1*, vary substantially among ethnic groups, region-specific data are essential for tailoring pharmacogenomic recommendations to local populations [30]. In Central and South Asian regions, including Kazakhstan, such data remain sparse, underscoring the importance of national and multicenter initiatives to establish population-specific pharmacogenomic profiles [31].

Another challenge lies in the operational and infrastructural aspects of implementation. Routine pharmacogenomic testing requires access to certified molecular diagnostic laboratories, trained personnel, and standardized reporting systems [32]. Integrating genomic results into electronic medical records and clinical decision-support systems would allow real-time guidance for oncologists during prescribing [33]. Furthermore, establishing clear protocols for sample collection, testing turnaround times, and quality assurance is vital to ensure reliability and clinical utility. Collaboration between clinical pharmacologists, geneticists, oncologists, and bioinformaticians is essential for achieving this goal.

Economic considerations also play a significant role in limiting adoption, particularly in low- and middle-income countries (Table 2). Although the cost of genotyping has declined markedly in recent years, resource constraints often hinder its routine use. Cost-effectiveness studies have shown that preemptive testing for *TPMT* or *DPYD* can prevent severe, hospitalization-requiring toxicities and reduce overall treatment costs, yet upfront investment in infrastructure and training remains necessary [34]. In this context, pilot programs and targeted testing for high-risk drug–gene pairs may serve as feasible starting points for wider implementation.

Equally important are the ethical and educational dimensions of pharmacogenomics integration. Genetic testing in minors requires careful consideration of consent, data privacy, and potential psychosocial implications for families. Clinicians must also be adequately trained to interpret and communicate genomic results in an understandable and actionable manner. Incorporating pharmacogenomics into medical education and oncology residency programs could strengthen clinician confidence and promote appropriate use of testing results in clinical practice [35].

To accelerate progress, national health systems and research programs should prioritize the establishment of centralized pharmacogenomic databases linked to cancer registries. Such platforms can support the collection and analysis of large-scale genotype–phenotype data, facilitate discovery of new markers, and enable the integration of artificial intelligence and machine learning tools to predict toxicity or treatment response. These developments would not only improve individualized patient care but also strengthen public health strategies for pediatric oncology at the population level.

**Table 2:** Key aspects, challenges, and strategies for implementing pharmacogenomic testing in pediatric oncology.

Implementation Domain	Key Issues/Barriers	Examples and Evidence	Proposed Solutions/ Strategies	Ref.
Clinical Validation and Guidelines	Limited number of pharmacogenes with strong clinical evidence	CPIC and DPWG guidelines: *TPMT* and *NUDT15* (thiopurines), *DPYD* (fluoropyrimidines), *CYP2D6* (tamoxifen)	Expand pediatric-specific validation studies; harmonize international dosing guidelines	[36]
Population Diversity and Genetic Variability	Lack of large, ethnically diverse pediatric cohorts; regional allele frequency differences	Variants such as *NUDT15*, *SLCO1B1*, *GSTA1* vary among populations; limited data in Central and South Asia	Establish national and multicenter studies to build population-specific pharmacogenomic databases	[37]
Infrastructure and Operational Capacity	Limited access to certified molecular laboratories and standardized workflows	Variability in sample handling, test turnaround times, and data reporting	Develop centralized genomic testing centers; integrate results into electronic health records and clinical decision-support systems	[38]
Economic and Resource Constraints	High initial costs of infrastructure and personnel training despite decreasing genotyping costs	Preemptive testing for *TPMT* and *DPYD* shown to prevent severe toxicities and reduce long-term costs	Initiate pilot projects focused on high-risk gene–drug pairs; seek government and institutional funding	[39]
Ethical and Legal Considerations	Informed consent in minors; data privacy and psychosocial implications for families	Parental consent and interpretation challenges in pediatric settings	Establish ethical frameworks and data governance policies; ensure transparency and family counseling	[40]
Education and Professional Training	Limited genetic literacy among clinicians and oncology staff	Clinicians may struggle to interpret or apply genomic data	Integrate pharmacogenomics into medical and pharmacy curricula; provide continuous professional development	[41]
Data Integration and Innovation	Fragmented data systems and lack of real-time clinical support	Absence of linked databases and decision-support tools	Create centralized pharmacogenomic registries linked to cancer databases; apply AI/ML models for predictive analytics	[42]

## 4. Emerging Approaches

The rapid evolution of genomic technologies and data science is reshaping the landscape of pharmacogenomics, offering unprecedented opportunities to refine cancer therapy in children. Moving beyond single-gene associations, current research increasingly embraces multi-omics, systems biology, and artificial intelligence (AI) to capture the complex molecular interactions that govern drug response, toxicity, and disease progression. These emerging approaches promise to close the translational gap between genetic discovery and clinical application, bringing the vision of precision paediatric oncology closer to reality.

Advances in multi-omics profiling, encompassing genomics, transcriptomics, proteomics, metabolomics, and epigenomics, allow a more comprehensive characterization of drug–gene–environment interactions. In paediatric oncology, such integrative analyses can reveal new biomarkers that predict treatment response or identify molecular pathways responsible for resistance and toxicity [43]. For example, combining genomic variants with transcriptomic and metabolomic data has been shown to enhance the prediction of methotrexate clearance and thiopurine-induced myelosuppression [44]. Similarly, epigenetic markers such as DNA methylation patterns and microRNA expression profiles are emerging as modifiers of chemotherapeutic sensitivity. These findings underscore the value of a holistic systems-pharmacology approach, in which multiple biological layers are analysed simultaneously to understand drug action within the broader cellular network rather than through isolated pathways [45].

The systems pharmacology paradigm integrates pharmacogenomic data with pharmacokinetic and pharmacodynamic (PK/PD) modelling to simulate how drugs behave in individual patients [46]. Computational models incorporating genetic, biochemical, and physiological variables can predict dose–response relationships and toxicity thresholds more accurately than traditional empirical methods. This approach is particularly relevant in paediatric oncology, where developmental changes in organ function, enzyme maturation, and drug transport profoundly affect pharmacokinetics. Incorporating genetic and age-dependent parameters into mechanistic PK/PD models can help refine dose individualization and improve the therapeutic index of anticancer agents in children [47].

At the intersection of big data and clinical practice, artificial intelligence and machine learning (ML) are emerging as transformative tools for pharmacogenomic research. AI algorithms can process complex, multidimensional datasets derived from genomic sequencing, clinical records, and laboratory data to identify hidden patterns and predictive markers of toxicity or response. For instance, ML-based models trained on large pediatric oncology cohorts could stratify patients by predicted risk of chemotherapy-induced cardiotoxicity, neurotoxicity, or myelosuppression, allowing clinicians to personalize therapy proactively [48]. Furthermore, AI-driven decision-support systems can integrate pharmacogenomic test results directly into electronic health records, providing real-time dosing recommendations and flagging potential drug–gene interactions at the point of care [49]. Such tools not only enhance clinical accuracy but also reduce the cognitive and administrative burden on healthcare providers.

The growing availability of high-throughput sequencing and cloud-based data platforms also enables the establishment of integrated pharmacogenomic registries. These digital infrastructures can link clinical, molecular, and pharmacologic data across institutions and regions, facilitating population-level analyses and accelerating the validation of genetic markers in diverse paediatric cohorts [50]. When combined with AI-based analytics, these registries can generate predictive models for treatment outcomes, toxicity risk, and long-term survivorship, thereby transforming raw genomic data into actionable clinical intelligence [51]. For countries developing national paediatric oncology programs, such as Kazakhstan, these systems represent a strategic opportunity to enhance data-driven decision-making, support translational research, and improve equity in access to precision medicine.

Looking forward, ethical and regulatory frameworks must evolve alongside technological progress to ensure the responsible use of genomic and AI technologies in children. Issues of data privacy, informed consent, and algorithmic transparency require particular attention, especially when dealing with minors and vulnerable populations. Collaboration among clinicians, data scientists, bioethicists, and policymakers will be essential to establish standards for data governance, interoperability, and clinical validation of AI-based tools.

## 5. Challenges in Clinical Implementation

Despite a growing body of evidence establishing pharmacogenomics as a cornerstone of precision oncology, the transition from research discovery to routine clinical practice remains challenging. These barriers are multifaceted (scientific, infrastructural, economic, ethical, and educational), and are particularly evident in paediatric oncology, where patient populations are smaller, treatment regimens are highly standardized, and ethical considerations demand heightened vigilance. Addressing these challenges is essential to ensure that pharmacogenomic advances are effectively translated into meaningful clinical benefits for children with cancer.

One of the most significant challenges is the limited availability of robust clinical evidence demonstrating clear cost-effectiveness and clinical utility of pharmacogenomic testing in paediatric populations. While strong data exist for specific gene–drug pairs such as *TPMT* and *NUDT15* in thiopurine metabolism, many proposed associations remain unvalidated or lack replication in large, ethnically diverse cohorts [9]. Small sample sizes, heterogeneous treatment protocols, and the rarity of certain paediatric malignancies hinder the establishment of definitive genotype–phenotype correlations. As a result, clinicians may hesitate to modify established chemotherapy protocols based on genetic information that lacks universally accepted clinical thresholds or guidelines.

The economic burden of implementing pharmacogenomic testing further complicates widespread adoption, especially in low- and middle-income countries. Although the cost of genotyping and sequencing has declined substantially, the infrastructure required to support routine testing, including laboratory facilities, bioinformatics platforms, and trained personnel, remains resource-intensive [27,29]. Health systems operating under constrained budgets often prioritize immediate treatment access over genomic screening programs, despite the long-term cost savings associated with preventing severe toxicity and hospitalization. Economic evaluations that quantify the financial and clinical benefits of targeted pharmacogenomic testing are crucial for policy advocacy and reimbursement inclusion.

From an infrastructural standpoint, the successful implementation of pharmacogenomics depends on the establishment of integrated molecular diagnostics and informatics networks. Many paediatric oncology centres lack on-site genetic testing capabilities or standardized pipelines for sample collection, sequencing, data interpretation, and clinical reporting [52,53]. Furthermore, the absence of interoperable electronic health records limits the ability to integrate genomic data into clinical workflows. Developing centralized, accredited laboratories and secure, cloud-based databases, linked to hospital information systems, would facilitate rapid data exchange, improve quality control, and ensure uniform standards of practice [54]. Collaborative national or regional networks could also pool data across institutions, increasing statistical power for rare variants and enhancing the discovery of population-specific markers.

Ethical and legal considerations represent another critical domain of concern. The use of genetic testing in children raises complex issues regarding informed consent, data ownership, privacy, and the potential psychosocial impact of revealing heritable risk factors [55]. Unlike adult patients, minors cannot provide independent consent, and parents or guardians may differ in their understanding of the implications of genetic findings. Moreover, pharmacogenomic data collected during cancer treatment may have future relevance for other family members, raising questions about the disclosure and sharing of such data [56]. Establishing clear ethical guidelines and robust governance frameworks is essential to ensure the responsible use of genomic information while protecting patient rights and confidentiality.

Moreover, the educational challenge extends beyond clinicians to include laboratory technicians, bioinformaticians, and genetic counsellors who are essential for the accurate implementation and interpretation of PGx testing. Medical and pharmacy curricula are often already crowded, leaving limited room for in-depth PGx education. Additionally, not all pharmacology departments have PGx experts capable of developing context-specific teaching materials. This gap in training at both the diagnostic and clinical levels poses a significant barrier to the widespread adoption of PGx, particularly in resource-limited settings where access to specialised training is scarce. Addressing these educational deficiencies requires coordinated efforts across academic institutions, professional societies, and healthcare systems to integrate PGx into foundational and continuing education programs.

In addition, the lack of standardized international and national guidelines for pharmacogenomic testing in paediatric oncology contributes to variability in clinical practice.

## 6. Discussion and Future Directions

**Figure 1:**
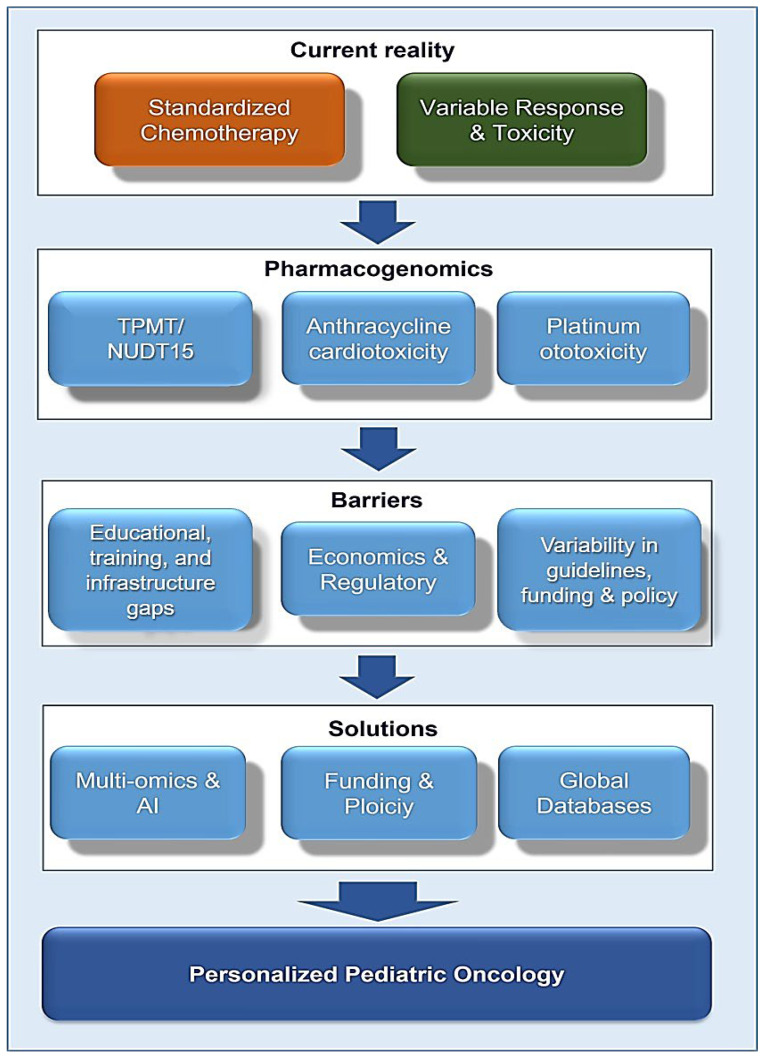
Integration of pharmacogenomics insights into clinical practice and personalized therapy.

Advances in genomic science have revealed how inherited and somatic genetic variations influence the absorption, metabolism, and response to antineoplastic agents, explaining much of the inter-individual variability in treatment efficacy and toxicity observed among children with cancer [57]. The systematic integration of these genetic insights into clinical practice holds the key to improving therapeutic precision, reducing preventable adverse events, and ultimately enhancing both survival and quality of life for paediatric patients (Figure 1).

Across multiple classes of chemotherapeutic agents, such as thiopurines, methotrexate, anthracyclines, alkylating agents, and platinum compounds, specific pharmacogenetic variants have been identified that predict treatment response and toxicity risk [58]. The clinical implementation of genotyping for *TPMT* and *NUDT15* has already demonstrated how pharmacogenomic testing can meaningfully guide dose adjustment and minimise severe myelosuppression [59] (Figure 1). Similar progress is being made in understanding the genetic determinants of methotrexate clearance, anthracycline-induced cardiotoxicity, and platinum-associated ototoxicity. Collectively, these findings underscore the tangible benefits of integrating genomic data into therapeutic decision-making for childhood malignancies.

Nevertheless, significant challenges remain. Implementation is constrained by limited infrastructure, insufficient paediatric-specific evidence, variability in clinical guidelines, economic and ethical barriers, and significant educational gaps in the current healthcare workforce. To move beyond isolated applications, pharmacogenomic testing must become an integral component of standardised paediatric oncology protocols [60]. This requires investment in molecular diagnostic laboratories, secure and interoperable data systems, and training programs that equip clinicians with the necessary skills to interpret and act upon genomic information. Equally crucial is the establishment of regionally relevant pharmacogenomic databases, which will help ensure that clinical recommendations reflect the genetic diversity of local populations.

Emerging technologies such as multi-omics integration, systems pharmacology, and artificial intelligence provide an unprecedented opportunity to accelerate translation from bench to bedside. By combining genomic, transcriptomic, and clinical data through predictive computational models, researchers and clinicians can identify complex biomarker networks that inform dosing strategies and toxicity prevention with greater precision. The development of AI-powered decision-support tools embedded within electronic health records can further facilitate real-time, evidence-based treatment adjustments, bridging the gap between genetic discovery and bedside application.

Policy frameworks must evolve in parallel with these scientific advances. National health systems should recognize pharmacogenomics as an essential element of high-quality paediatric oncology care and allocate dedicated funding to support implementation. Collaborative international initiatives can accelerate data sharing, standardization, and capacity building, ensuring equitable access to genomic medicine across different healthcare settings. Ethical oversight must also remain a priority, safeguarding patient autonomy, data privacy, and the responsible use of genomic information, particularly in vulnerable paediatric populations. The successful integration of pharmacogenomics into paediatric oncology demands a multidimensional approach, combining scientific innovation, clinical translation, infrastructure development, and policy reform. As genomic medicine continues to mature, its promise extends beyond improving individual treatment outcomes to reshaping the entire paradigm of paediatric cancer care. By investing in the systems, knowledge, and equity required to support pharmacogenomic implementation, healthcare systems can advance toward a future in which every child with cancer receives therapy that is not only effective but also genetically and ethically tailored to ensure the best possible outcome.

Furthermore, educational initiatives must be prioritised to ensure that clinicians, pharmacists, and laboratory staff are proficient in PGx principles and practices. This includes revising medical and pharmacy curricula, offering specialised training modules, and fostering interdisciplinary collaboration between genetics and oncology specialities.

## 7. Conclusions

Pharmacogenomics stands at the forefront of precision medicine in paediatric oncology, bridging the gap between genetic discovery and individualised therapy. By elucidating how germline and somatic genetic variants shape the metabolism, distribution, and activity of chemotherapeutic drugs, pharmacogenomic insights can dramatically reduce preventable toxicity and improve therapeutic efficacy. Despite compelling evidence for several validated gene–drug pairs, such as TPMT and NUDT15 in thiopurine metabolism, clinical implementation remains uneven due to infrastructural, economic, and regulatory challenges, particularly in resource-limited settings. Moving forward, the success of pharmacogenomics in paediatric oncology will depend on a multifaceted strategy encompassing scientific, clinical, and policy domains. Investment in molecular diagnostic infrastructure, data integration platforms, and clinician training is essential for routine adoption. The development of regionally representative pharmacogenomic databases will ensure that therapeutic recommendations reflect local genetic diversity. At the same time, emerging technologies such as multi-omics profiling, systems pharmacology, and AI-based predictive modelling offer powerful tools to refine risk stratification and optimize treatment design. Ultimately, embedding pharmacogenomic testing within standardized paediatric oncology protocols represents a critical step toward safer and more effective cancer care. Through collaborative global and national initiatives that integrate genomics, ethics, and equitable access, pharmacogenomics can fulfil its promise: delivering individualized, evidence-based, and humane therapy for every child with cancer.

## Data Availability

Not applicable.

## References

[1] Chen X., Yang W., Roberts C.W.M., Zhang J. (2024). Developmental origins shape the paediatric cancer genome. Nat. Rev. Cancer.

[2] Orbach D., Brecht I.B., Corradini N., Bouchoucha Y., Roganovic J., Bourdeaut F., Reguerre Y., Kuiper R.P., Bressac de Paillerets B., Ferrari A. (2023). The role of cancer predisposition syndrome in children and adolescents with very rare tumours. EJC Paediatr. Oncol..

[3] Coluzzi F., Di Stefano G., Scerpa M.S., Rocco M., Di Nardo G., Innocenti A., Vittori A., Ferretti A., Truini A. (2025). The Challenge of Managing Neuropathic Pain in Children and Adolescents with Cancer. Cancers.

[4] Liu Y., Zhang Y., Li H., Hu T.Y. (2025). Recent advances in the bench-to-bedside translation of cancer nanomedicines. Acta Pharm. Sin. B.

[5] Qahwaji R., Ashankyty I., Sannan N.S., Hazzazi M.S., Basabrain A.A., Mobashir M. (2024). Pharmacogenomics: A Genetic Approach to Drug Development and Therapy. Pharmaceuticals.

[6] Sánchez-Bayona R., Catalán C., Cobos M.A., Bergamino M. (2025). Pharmacogenomics in Solid Tumors: A Comprehensive Review of Genetic Variability and Its Clinical Implications. Cancers.

[7] Tremmel R., Hübschmann D., Schaeffeler E., Pirmann S., Fröhling S., Schwab M. (2025). Innovation in cancer pharmacotherapy through integrative consideration of germline and tumor genomes. Pharmacol. Rev..

[8] Wu X., Xiong H. (2024). The Role of Pharmacogenetic-Based Pharmacokinetic Analysis in Precise Breast Cancer Treatment. Pharmaceutics.

[9] Lubis I.S., Anggadiredja K., Artarini A.A., Sari N.M., Suryawan N., Zazuli Z. (2025). NUDT15 Pharmacogenetics in Acute Lymphoblastic Leukemia: Synthesizing Progress for Personalized Thiopurine Therapy. Med. Sci..

[10] Du S., Huang X., He X., Mao M., Chen M., Zhang R., Shao H., Lv Z., Liu X., Chuan J. (2023). Association of *NUDT15* gene polymorphism with adverse reaction, treatment efficacy, and dose of 6-mercaptopurine in patients with acute lymphoblastic leukemia: A systematic review and meta-analysis. Haematologica.

[11] Guo Q., Sun J.-L., Li R., Li X. (2024). Involvement of the ABCB1 C3435T Variant but Not the MTHFR C677T or MTHFR A1298C Variant in High-Dose Methotrexate-Induced Toxicity in Pediatric Acute Lymphoblastic Leukemia Patients in China. Int. J. Gen. Med..

[12] Krüger-Genge A., Köhler S., Laube M., Haileka V., Lemm S., Majchrzak K., Kammerer S., Schulz C., Storsberg J., Pietzsch J. (2023). Anti-Cancer Prodrug Cyclophosphamide Exerts Thrombogenic Effects on Human Venous Endothelial Cells Independent of CYP450 Activation—Relevance to Thrombosis. Cells.

[13] El-Serafi I., Steele S., Mudigonda K. (2024). Cyclophosphamide Pharmacogenomic Variation in Cancer Treatment and Its Effect on Bioactivation and Pharmacokinetics. Adv. Pharmacol. Pharm. Sci..

[14] Nguyen A.-H., Biswas M., Puangpetch A., Prommas S., Pakakasama S., Anurathapan U., Rachanakul J., Sukprasong R., Nuntharadtanaphong N., Jongjitsook N. (2022). Effect of GSTA1 Variants on Busulfan-Based Conditioning Regimen Prior to Allogenic Hematopoietic Stem-Cell Transplantation in Pediatric Asians. Pharmaceutics.

[15] Ryan T.D., Bates J.E., Kinahan K.E., Leger K.J., Mulrooney D.A., Narayan H.K., Ness K., Okwuosa T.M., Rainusso N.C., Steinberger J. (2025). Cardiovascular Toxicity in Patients Treated for Childhood Cancer: A Scientific Statement from the American Heart Association. Circulation.

[16] Spadafora L., Di Muro F.M., Intonti C., Massa L., Monelli M., Pedretti R.F.E., Palazzo Adriano E., Guarini P., Cantiello G., Bernardi M. (2025). Lifestyle and Pharmacological Interventions to Prevent Anthracycline-Related Cardiotoxicity in Cancer Patients. J. Cardiovasc. Dev. Dis..

[17] Wong-Siegel J.R., Kim Y., Stitziel N.O., Javaheri A. (2023). Genetic Testing in Evaluating Risk of Anthracycline Cardiomyopathy. JACC CardioOncol..

[18] Tay N., Laakso E.L., Schweitzer D., Endersby R., Vetter I., Starobova H. (2022). Chemotherapy-induced peripheral neuropathy in children and adolescent cancer patients. Front. Mol. Biosci..

[19] Mufti K., Cordova M., Scott E.N., Trueman J.N., Lovnicki J.M., Loucks C.M., Rassekh S.R., Ross C.J.D., Carleton B.C., Groeneweg G.S.S. (2024). Genomic variations associated with risk and protection against vincristine-induced peripheral neuropathy in pediatric cancer patients. NPJ Genom. Med..

[20] Romano A., Attinà G., Maurizi P., Talloa D., Mastrangelo S., Ruggiero A. (2025). Platinum-induced ototoxicity and hearing impairment in children and adolescents. Drugs Context.

[21] Díaz-Villamarín X., Fernández-Varón E., Rojas Romero M.C., Callejas-Rubio J.L., Cabeza-Barrera J., Rodríguez-Nogales A., Gálvez J., Morón R. (2023). Azathioprine dose tailoring based on pharmacogenetic information: Insights of clinical implementation. Biomed. Pharmacother..

[22] Zhao X., Wu P., Yang Z., Miao R.-R. (2024). Relationship between the efficacy and adverse effects of methotrexate and gene polymorphism. Egypt. J. Med. Hum. Genet..

[23] Berkman A.M., Hildebrandt M.A.T., Landstrom A.P. (2021). The genetic underpinnings of anthracycline-induced cardiomyopathy predisposition. Clin. Genet..

[24] Shalaby N., Zaki H.F., Badary O.A., Kamal S., Nagy M., Makhlouf D., Elnashar A., Elnadi E., Abdelshafi S.A., Abouelnaga S. (2024). Efficacy and Toxicity of Vincristine and CYP3A5 Genetic Polymorphism in Rhabdomyosarcoma Pediatric Egyptian Patients. Asian Pac. J. Cancer Prev..

[25] Powles T., Csoszi T., Loriot Y., Matsubara N., Geczi L., Cheng S.Y., Fradet Y., Alva A., Oudard S., Vulsteke C. (2025). Cisplatin- or Carboplatin-Based Chemotherapy Plus Pembrolizumab in Advanced Urothelial Cancer: Exploratory Analysis From the Phase 3 KEYNOTE-361 Study. Clin. Genitourin. Cancer.

[26] Cooper J., Pratt J., Park J., Fahim C., Lovnicki J.M., Groeneweg G.S.S., Carleton B., Straus S. (2024). Implementation of pharmacogenetic testing in pediatric oncology: Barriers and facilitators assessment at eight Canadian academic health centres. Pharmacogenomics J..

[27] Díaz-Villamarín X., Martínez-Pérez M., Nieto-Sánchez M.T., Fernández-Varón E., Torres-García A., Blancas I., Cabeza-Barrera J., Morón R. (2025). Clinical Pharmacogenetics: Results After Implementation of Preemptive Tests in Daily Routine. J. Pers. Med..

[28] Maillard M., Nishii R., Yang W., Hoshitsuki K., Chepyala D., Lee S.H.R., Nguyen J.Q., Relling M.V., Crews K.R., Leggas M. (2024). Additive effects of TPMT and NUDT15 on thiopurine toxicity in children with acute lymphoblastic leukemia across multiethnic populations. JNCI J. Natl. Cancer Inst..

[29] Barker C.I.S., Groeneweg G., Maitland-van der Zee A.H., Rieder M.J., Hawcutt D.B., Hubbard T.J., Swen J.J., Carleton B.C. (2022). Pharmacogenomic testing in paediatrics: Clinical implementation strategies. Br. J. Clin. Pharmacol..

[30] Cacabelos R., Naidoo V., Corzo L., Cacabelos N., Carril J.C. (2021). Genophenotypic Factors and Pharmacogenomics in Adverse Drug Reactions. Int. J. Mol. Sci..

[31] Ranasinghe P., Sirisena N., Vishnukanthan T., Ariadurai J.N., Thilakarathne S., Priyadarshani C.D.N., Bhagya Hendalage D.P., Dissanayake V.H.W. (2024). Frequency of pharmacogenomic variants affecting efficacy and safety of anti-cancer drugs in a south Asian population from Sri Lanka. BMC Med. Genom..

[32] Mroz P., Michel S., Allen J.D., Meyer T., McGonagle E.J., Carpentier R., Vecchia A., Schlichte A., Bishop J.R., Dunnenberger H.M. (2021). Development and Implementation of In-House Pharmacogenomic Testing Program at a Major Academic Health System. Front. Genet..

[33] Morris S.A., Nguyen D.G., Morris V., Mroz K., Kwange S.O., Patel J.N. (2024). Integrating pharmacogenomic results in the electronic health record to facilitate precision medicine at a large multisite health system. JACCP J. Am. Coll. Clin. Pharm..

[34] Valdez-Acosta S., Zubiaur P., Casado M.A., Novalbos J., Casajús A., Campodónico D., Oyagüez I., Abad-Santos F. (2023). Preemptive TPMT Genotyping and Adherence to Genotype-Based Therapeutic Recommendations Reduces the Healthcare Cost in Patients Receiving Azathioprine or 6-Mercaptopurine for Autoimmune Diseases. J. Pers. Med..

[35] Thottunkal S., Spahn C., Wang B., Rohatgi N., Hong J., Khandelwal A., Palaniappan L. (2025). Clinician Experiences at the Frontier of Pharmacogenomics and Future Directions. J. Pers. Med..

[36] Rim J.H., Kim Y.-g., Kim S., Choi R., Lee J.-S., Park S., Lee W., Song E.Y., Lee S.-Y., Chun S. (2024). Clinical Pharmacogenetic Testing and Application: 2024 Updated Guidelines by the Korean Society for Laboratory Medicine. Ann. Lab. Med..

[37] Hamdani S., Hamijoyo L., Amalia R., Barliana M.I. (2025). Gene polymorphisms associated with immunosuppressant adverse effects in systemic lupus erythematosus: A narrative review. Front. Genet..

[38] Husereau D., Steuten L., Muthu V., Thomas D.M., Spinner D.S., Ivany C., Mengel M., Sheffield B., Yip S., Jacobs P. (2022). Effective and Efficient Delivery of Genome-Based Testing-What Conditions Are Necessary for Health System Readiness?. Healthcare.

[39] Wong L.Y.F., Sutcliffe A.G., Ho C.L.T., Lu Y., Williams C.L., Afzal F., Purkayastha M. (2025). Clinical and cost-effectiveness of pharmacogenomic testing for anthracycline-induced cardiotoxicity in childhood cancer: A systematic review and meta-analysis. Front. Pharmacol..

[40] Chen D. (2024). Ethical frameworks of informed consent in the age of pediatric precision medicine. Camb. Prism. Precis. Med..

[41] Bailey S.L., Messersmith D., Empey P.E. (2025). Pharmacogenomics education among professional societies: Assessing practices and future needs. Pharmacogenomics.

[42] Sadee W., Wang D., Hartmann K., Toland A.E. (2023). Pharmacogenomics: Driving Personalized Medicine. Pharmacol. Rev..

[43] Du P., Fan R., Zhang N., Wu C., Zhang Y. (2024). Advances in Integrated Multi-omics Analysis for Drug-Target Identification. Biomolecules.

[44] Vieujean S., Louis E. (2023). Precision medicine and drug optimization in adult inflammatory bowel disease patients. Ther. Adv. Gastroenterol..

[45] Song J., Yang P., Chen C., Ding W., Tillement O., Bai H., Zhang S. (2025). Targeting epigenetic regulators as a promising avenue to overcome cancer therapy resistance. Signal Transduct. Target. Ther..

[46] Tindall M.J., Cucurull-Sanchez L., Mistry H., Yates J.W.T. (2023). Quantitative Systems Pharmacology and Machine Learning: A Match Made in Heaven or Hell?. J. Pharmacol. Exp. Ther..

[47] Cheung S.Y.A., Hay J.L., Lin Y.-W., de Greef R., Bullock J. (2024). Pediatric oncology drug development and dosage optimization. Front. Oncol..

[48] Hashem H., Sultan I. (2025). Revolutionizing precision oncology: The role of artificial intelligence in personalized pediatric cancer care. Front. Med..

[49] Huang W., Wang X., Chen Y., Yu C., Zhang S. (2025). Advancing drug-drug interactions research: Integrating AI-powered prediction, vulnerable populations, and regulatory insights. Front. Pharmacol..

[50] McDermott J.H., Tsakiroglou M., Newman W.G., Pirmohamed M. (2025). Pharmacogenomics in the UK National Health Service: Progress towards implementation. Br. J. Clin. Pharmacol..

[51] Huhulea E.N., Huang L., Eng S., Sumawi B., Huang A., Aifuwa E., Hirani R., Tiwari R.K., Etienne M. (2025). Artificial Intelligence Advancements in Oncology: A Review of Current Trends and Future Directions. Biomedicines.

[52] Forrest S.J., Gupta H., Ward A., Li Y.Y., Doan D., Al-Ibraheemi A., Alexandrescu S., Bandopadhayay P., Shusterman S., Mullen E.A. (2024). Molecular profiling of 888 pediatric tumors informs future precision trials and data-sharing initiatives in pediatric cancer. Nat. Commun..

[53] Jezkova J., Shaw S., Taverner N.V., Williams H.J. (2022). Rapid genome sequencing for pediatrics. Hum. Mutat..

[54] Tariq S., Tariq S., Shoukat A.A. (2023). Centralized healthcare database for ensuring better healthcare: Are we lagging behind?. Pak. J. Med. Sci..

[55] Refolo P., Ferracuti S., Grassi S., Raimondi C., Mercuri G., Zedda M., Aulino G., Spagnolo A.G., Oliva A. (2025). Ethical issues in the use of genetic predictions of aggressive behavior in the criminal justice system: A systematic review. Front. Genet..

[56] Alkilani H.M., El-Akouri K., Farooq A., Shi Z., Al-Shafai M., Stotland M., Khodjet-El-khil H. (2025). Parental knowledge and attitudes toward genetic counseling and childhood genetic testing for congenital anomalies in Qatar. J. Genet. Couns..

[57] Jamalinia M., Weiskirchen R. (2025). Advances in personalized medicine: Translating genomic insights into targeted therapies for cancer treatment. Ann. Transl. Med..

[58] Elzagallaai A.A., Carleton B.C., Rieder M.J. (2021). Pharmacogenomics in Pediatric Oncology: Mitigating Adverse Drug Reactions While Preserving Efficacy. Annu. Rev. Pharmacol. Toxicol..

[59] Roberts C., Peters J., Sazonvos A., Goodman N., Sharip M., Smith R., Bishara M., Bewshea C., Lin S., Chanchlani N. (2025). Clinical Utility and Cost-Effectiveness of Pretreatment NUDT15 Pharmacogenetic Testing to Prevent Thiopurine-Induced Myelosuppression: A Genotype-First Reverse Phenotyping Cohort Study Within the UK NIHR Inflammatory Bowel Disease Bioresource. Aliment. Pharmacol. Ther..

[60] Leitch T.M., Killam S.R., Brown K.E., Katseanes K.C., George K.M., Schwanke C., Loveland J., Elias A.F., Haney K., Krebsbach K. (2022). Ensuring equity: Pharmacogenetic implementation in rural and tribal communities. Front. Pharmacol..

